# Single-molecule fluorescence-based analysis of protein conformation, interaction, and oligomerization in cellular systems

**DOI:** 10.1007/s12551-017-0366-3

**Published:** 2017-12-14

**Authors:** Kenji Okamoto, Michio Hiroshima, Yasushi Sako

**Affiliations:** 1 Cellular Informatics Laboratory, RIKEN, 2-1 Hirosawa, Wako, 351-0198 Japan; 2 Laboratory for Cell Signaling Dynamics, QBiC, RIKEN, 6-2-3, Fruedai, Suita, 565-0874 Japan

**Keywords:** Fluorescence resonance energy transfer (FRET), Reaction kinetics, Single-molecule imaging (SMI), Single-photon counting (SPC), Super-resolution microscope

## Abstract

Single-molecule imaging (SMI) of proteins in operation has a history of intensive investigations over 20 years and is now widely used in various fields of biology and biotechnology. We review the recent advances in SMI of fluorescently-tagged proteins in structural biology, focusing on technical applicability of SMI to the measurements in living cells. Basic technologies and recent applications of SMI in structural biology are introduced. Distinct from other methods in structural biology, SMI directly observes single molecules and single-molecule events one-by-one, thus, explicitly analyzing the distribution of protein structures and the history of protein dynamics. It also allows one to detect single events of protein interaction. One unique feature of SMI is that it is applicable in complicated and heterogeneous environments, including living cells. The numbers, location, movements, interaction, oligomerization, and conformation of single-protein molecules have been determined using SMI in cellular systems.

## Introduction

Single-molecule imaging (SMI; Sako et al. 2012; Iino et al. 2017) of proteins working in aqueous conditions was first reported in 1995 in the detection of ATP hydrolysis (Funatsu et al. 1995) and sliding movements (Sase et al. 1995) of the muscle actomyosin system. Shortly afterward, SMI was extended to the analysis of Förster resonance energy transfer between single pairs of fluorophore-labeled single-protein molecules (single-pair fluorescence resonance energy transfer; spFRET). spFRET was first used to determine the conformational dynamics of an enzymatic protein (Ha et al. 1999a). Thus, SMI was developed as a technique to study the functional and structural dynamics of protein molecules in vitro. With fluorescence optical microscopes, SMI is easily applicable to complicated biological reaction systems. Observations of fluorophores that are conjugated to single molecules in living cells were reported in 2000 (Sako et al. 2000; Shütz et al. 2000). Biological systems are highly complicated in structure and function. They are working with small numbers of reaction and low energy consumption. Single-molecule resolution is useful to study the mechanism of such biological systems.

SMI provides several types of information on the static and dynamic structure of proteins and other biological macromolecules in vitro and *in cellula*. spFRET between fluorophores that conjugated to different sites in a single-protein molecule (intra-molecular spFRET) is often used to detect the conformational transition or fluctuation in single molecules of proteins (Ha et al. 1999a; Kozuka et al. 2006; Hibino et al. 2009). In contrast, spFRET between fluorophores that are conjugated to disparate protein molecules (inter-molecular spFRET) allows one to detect the homo- or hetero-oligomerization and interaction of proteins (Sako et al. 2000; Nguyen et al. 2003). Colocalization analysis of multiple molecules is also used to measure protein oligomerization and interaction (Ulbrich and Isacoff 2007; Hiroshima et al. 2012). Although spFRET analysis has higher spatial resolution than colocalization analysis, occurrence of FRET depends on distance and orientation of the fluorophores which are difficult to control ad libitum. Fluorescence correlation spectroscopy (FCS), fluorescence cross-correlation spectroscopy (FCCS), and photon counting histogram (PCH) are not imaging but single-molecule detection technologies that generate structural information on proteins (Sako et al. 2012). Detecting the fluctuation of the fluorescence intensity caused by exchange of fluorophores in a fixed focus volume, FCS, FCCS, and PCH determine the lateral diffusion coefficient, which is a function of the effective radius, affinity between two colors of particles, and average and distribution of oligomerization size, respectively. These parameters reflect protein conformation and formation of protein complexes. FCCS and PCH setups are applicable for measuring FRET.

Based on optical microscopy, the spatial resolution of SMI and other single-molecule measurements is limited to several hundreds of nanometers. Even in FRET measurements, the typical detection length between fluorophores is 5 nm. Therefore, information about the distributions and dynamics in nanometer to sub-micrometer structures is obtained primarily by single-molecule measurements. In addition to its low spatial resolution, SMI suffers from several technical difficulties. A primordial problem is its requirement of fluorescence labeling which can deform target molecules. Control experiments using conventional biochemical methods are needed to verify the consistency between results with and without labeling. Instability in fluorescence emission and photobleaching of the fluorophores are the major factors limiting spatial accuracy, temporal resolution, and observation period. Data processing in SMI measurements is often time-consuming.

Despite these drawbacks, SMI provides several types of structural information difficult to obtain using other methods. The assessment of the structural distribution of single species of molecules and the dynamic history of an individual molecule are among the many possibilities with SMI measurements. The structure of proteins might not be constrained to a single conformation. Using NMR measurement, multiple conformations have been suggested for even small proteins including the RAS family GTPases (Ito et al. 1997). Based on recent data using in-cell NMR technology, a significant fraction of proteins in cells has been proposed to be unfolded (Inomata et al. 2009). Post-translational modification, such as phosphorylation and methylation, can cause significant conformational changes in proteins. As a simpler case of structural variance, the clustering number of proteins must assume a distribution. The detection of the structural distribution of proteins is important because protein function and activity are generally specific to a structure.

Moreover, single-protein molecules might shuttle between different conformations over time. It is possible that the conformational drifting is not a simple Markov process (Lu et al. 1998; Edman and Riglar 2000; Morimatsu et al. 2007). Tracing the history of single-molecule structures and reactions is indispensable for detecting such complex dynamics. One useful feature of SMI is that it does not require synchronization with regard to measuring kinetics. At equilibrium or in the steady state, the fraction of each conformation does not change globally with time, rendering kinetic measurement in ensemble of molecules difficult. However, each molecule undergoes this conformational transition continually, which can be detected using SMI. This property of SMI is good for tracking non-Markov processes that require statistical analysis in the steady state. In SMI measurements, reactions of different molecules can be synchronized virtually by superimposing the starting points of individual reaction events, then we can analyze the lifetime distribution of each state.

The synchronization of multiple molecules, which is performed for kinetic or dynamic measurements of ensemble molecules, is usually difficult in living cells and in later stages of multi-step reaction systems, but these systems are amenable to asynchronous kinetic measurements in SMI. The function of proteins in living cells can be determined from movements, spatial distribution, and association/dissociation kinetics measured using SMI. Because these observations do not disturb structural measurements that are based on spFRET or fluorescence intensity, we can obtain detailed information on function–structure relationships by using SMI, even in complicated systems, such as living cells. In the following sections, we introduce several recent application of SMI in studies on protein structure in cellular systems. Readers may look up the recent special issues on SMI for further information (Orrit et al. 2014; Biteen and Willets 2017).

## Single-molecule FRET measurement

FRET is extensively used to investigate the structure of biomolecules. FRET occurs between two fluorescent dyes (fluorophores) that are in close proximity. When the donor dye is photo-excited, part of its energy is nonradiatively transferred to the acceptor dye, which consumes it to emit a fluorescence photon. The energy transfer efficiency *E*_FRET_ varies with the sixth power of interdye distance *r* expressed as $$ {E}_{FRET}=\frac{1}{1+{\left(\raisebox{1ex}{$r$}\!\left/ \!\raisebox{-1ex}{${R}_0$}\right.\right)}^6} $$, where *R*_0_ is the Förster distance, at which *E*_FRET_ = 0.5, and which changes drastically in the single-nanometer region. *E*_*FRET*_ also depends on orientation between two dyes; however, in many cases, rotation of dyes during the measurement allows averaging of the orientation factor. *E*_FRET_ can be measured experimentally as the ratio of fluorescence intensities of two dyes. When two dyes are conjugated to a single protein, the intramolecular FRET depends on the structure of the labeled protein and varies depending on its conformational change. In particular, spFRET measurements enable one to investigate the conformational dynamics of individual molecules.

## Structural measurements using in-solution spFRET

**Fig. 1:**
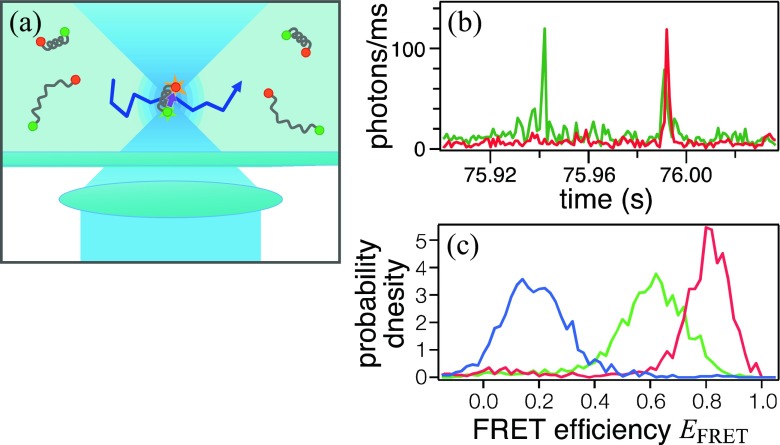
**a** Principle of in-solution spFRET measurement. Fluorescence is detected only from a single molecule diffusing through the microscope focus. **b** Example of fluorescence signals for donor (*green*) and acceptor (*red*). Single molecules are detected as bursts of fluorescence. **c** Example of spFRET histogram reconstructed from burst signals. Double-helix DNA labeled with interdye distance of 4 (*red*), 14 (*green*) and 24 (*blue*) base-pairs form single peaks (photobleached molecules are excluded by ALEX technique)

One typical measurement using spFRET is the detection of fluorescence from single molecules that are diffusing in solution. When excitation light is focused into solution, only molecules in the focus emit fluorescence (Fig. 1a). If the concentration is sufficiently low such that the average number of molecules in the focus is less than one, fluorescence from a single molecule is detected. For this type of experiments, a confocal microscope is used, usually with single-photon counting (SPC) detectors, such as photomultiplier tubes and avalanche photodiodes. Because a single molecule stays in focus only for a short period, typically a few milliseconds in an aqueous solution, it is detected as a burst of fluorescence photons (Fig. 1b). If the molecules are labeled with a pair of FRET dyes, *E*_FRET_ can be calculated for each molecule from the fluorescence photon counts of the two dyes in each burst.

In-solution spFRET measurements can be used to capture the structural distribution of a protein (PCH of *E*_*FRET*_; Fig. 1c; Fries et al. 1998; Deniz et al. 1999; Dahan et al. 1999). Different structural species form disparate peaks in a single-molecule *E*_FRET_ histogram. Although *E*_FRET_ in solution can be measured in bulk, it merely yields an average of the ensemble, i.e. one cannot distinguish whether all molecules have the same *E*_FRET_ or molecules with different *E*_FRET_ values are mixed. If the molecular structure changes within a burst of an spFRET measurement, *E*_FRET_ is detected between peaks of single structures. Therefore, the shape of the histogram depends on the relative rate of conformational dynamics against the burst duration (Schuler et al. 2001; Gopich and Szabo 2007; Tomov et al. 2012).

Advanced techniques, such as alternative laser excitation (ALEX; Kapanidis et al. 2004) have been developed. ALEX uses two colors of excitation lights, which alternately excite the donor and acceptor at high frequency, such that four types of photon counts, with two excitation colors and two detection colors, are detected separately. Then, in addition to *E*_FRET_, the quantity of stoichiometry *S*, which is defined as the labeling ratio between the donor and acceptor on single molecules, is calculated for each burst. Information on *S* enables one to distinguish bursts with a partially photobleached dye pair and exclude it from the statistics.

ALEX has also been used to examine the oligomerization state of proteins. For example, the ubiquitous recombination mediator RecR in *D. radiodurans* (drRecR) is thought to form stable homodimers and may form homotetramers. Kim et al. (2012) prepared drRecR monomers that were fluorescently labeled with a donor or acceptor to the artificially induced cysteine at the position of Q133 (Q133C) and found using ALEX that drRecR only formed dimers, not tetramers, in a mixed solution. They also observed that the N-terminus of the drRecR monomer is its dimerization interface, by comparing the ALEX results of drRecR with different labeling positions (Q133C and R37C), and that drRecR forms tetramers only in the presence of the drRecO protein, which links the C-termini of drRecR homodimers. spFRET measurement at sub-nanomolar concentration allowed the avoiding of bias from transient interaction in higher concentrations of proteins.

Another advantage of spFRET is the ability to examine the structure of intrinsically disordered proteins (IDPs). Because IDPs are natively unfolded and do not have fixed higher-order structures, their structures are unable to be measured using common methods, such as X-ray crystallography. In-solution spFRET can capture the average *E*_FRET_ of a fluctuating structure and reveal, for example, its dependence on the concentration of the denaturant or ionic strength of the solvent (Müller-Späth et al. 2010; Milles et al. 2012; Soranno et al. 2014; Wuttke et al. 2014). Application to in-cell measurement has also been reported (König et al. 2015), constituting a promising use for spFRET as achieved for FC(C)S (Pack et al. 2014).

## spFRET imaging of protein structures in cells

SMI using high-sensitivity cameras is more common than the SPC detection used in the spFRET measurement in solution. spFRET can be detected simultaneously from multiple molecules by using dual-color SMI. Because diffusing molecules in liquid move too rapidly to image, spFRET imaging and other SMI experiments are typically applied for in vitro systems in which sample molecules are immobilized on a glass substrate surface (Dickson et al. 1997; Lu et al. 1998; Ha et al. 1999b, 2002; Boukobza et al. 2001). spFRET imaging was also successful in live cells (Sako et al. 2000; Murakoshi et al. 2004; Sakon and Weninger 2010). Hibino et al. (2009) performed spFRET imaging of the cytoplasmic serine/threonine kinase RAF on the plasma membrane of cells. RAF is an effector of the membrane protein RAS. RAS is inactive in its GDP-bound form (RAS-GDP) and becomes activated through GDP/GTP exchange. The active GTP-bound form of RAS (RAS-GTP) interacts with downstream effectors, including RAF. With RAF which was genetically labeled with green fluorescent protein (GFP) and yellow fluorescent protein, Hibino et al. observed separate signals from those of single RAF molecules that were bound to RAS on the plasma membrane of living HeLa cells. A difference in *E*_FRET_ values obtained before and after cell stimulation with epidermal growth factor (EGF) was detected, indicating that RAF interacts with the inactive RAS-GDP and active RAS-GTP in the high-*E*_FRET_ (closed) and low-*E*_FRET_ (open) form, respectively.

## Time series analysis

spFRET measurements can trace the dynamics of individual molecules and allow one to analyze, for example, the paths or rates of conformational changes. To do so, spFRET time series are obtained. In SMI measurements of spFRET, *E*_*FRET*_ time series of single molecules are reconstructed from the brightness of fluorescence spots in each frame of a dual-color movie of donor and acceptor channels. Because many single molecules can be measured simultaneously, high-throughput measurements are possible in SMI. In contrast, because the microscope focus is fixed on the target molecule, only a single molecule can be measured at a time, and the molecule must be immobilized during the measurement based on SPC. Conversely, analysis with SPC is precise, because photon statistics are preserved in SPC signals, e.g., detection time of every photon can be recorded. In both types of measurement, data analysis is important because the signal-to-noise ratio in single-molecule detection is usually low. The hidden Markov model (HMM; McKinney et al. 2006; Bronson et al. 2009; Liu et al. 2010) is powerful in recovering the state transition trajectories from spFRET time series under the assumption of discrete states and a simple Markov process for transitions between them. The HMM has been extended to data analysis in SPC measurements (Okamoto and Sako 2012; Pirchi et al. 2016) and of the single-molecule non-Markov process (Zheng and Brown 2004; Sultana et al. 2013; Xue et al. 2015).

**Fig. 2:**
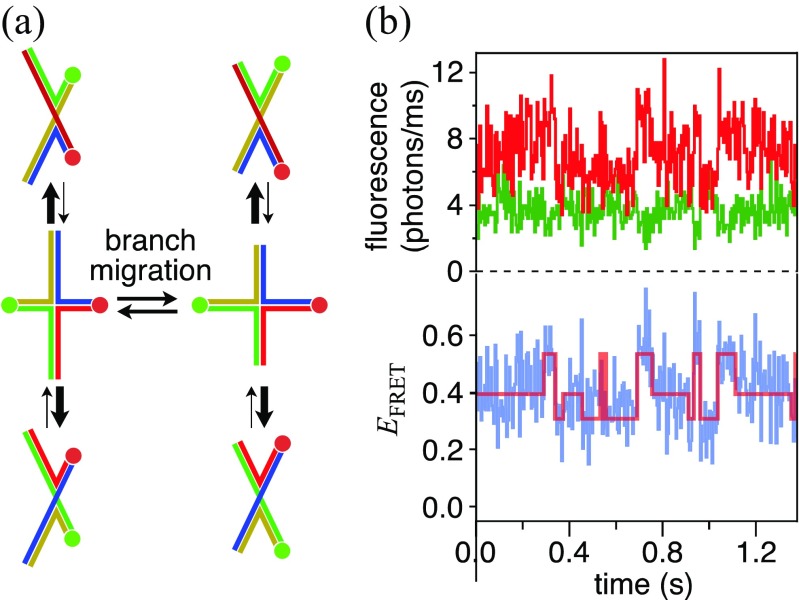
**a** The HJ is a four-way junction structure of DNA and assumes stacked (*top* and *bottom*) and extended (*middle*) structures. Branch migration is the movement of the crossing point during transition between extended structures. The *green* and *red circles* represent the donor and acceptor dye, respectively. **b** spFRET measurement results of branch migration between three states. FRET trajectory (*blue*) was calculated from fluorescence intensities (*green* for donor, *red* for acceptor). *Red line in the bottom panel* is the state transition trajectory obtained by HMM analysis

Although various measurement and analysis methods have been developed for spFRET experiments, a common reference sample for measuring spFRET dynamics is the Holliday junction (HJ). The HJ is a four-way junction structure that is composed of four DNA strands and possesses stacked and extended structures (Fig. 2a). The crossing point can move by rearrangement of base-pairs near the junction, termed “branch migration”. Because the HJ is easy to treat due to the stability of DNA, it is suitable as a reference for measuring molecular dynamics. Moreover, the rate of the conformational dynamics of HJ can be controlled by the Mg^2+^ concentration in solution (Panyutin et al. 1995). Several groups have reported a flip–flop motion between two stacked structures, reflecting simple two-state transitions with a large change in *E*_FRET_ (McKinney et al. 2006; Pirchi et al. 2016), and others have succeeded in resolving spontaneous branch migration, which is the transition between multiple states with relatively small difference in *E*_FRET_ (Fig. 2b; Karymov et al. 2008; Okamoto and Sako 2016).

spFRET time series measurements have been applied to protein systems. One typical target is the folding–unfolding dynamics of relatively small proteins. Labeling both ends of a polypeptide with dyes often shows minimal effects on the structure and function of proteins, allowing physiological changes in *E*_FRET_ to be detected (Jia et al. 1999; Talaga et al. 2000; Deniz et al. 2000; Schuler and Eaton 2008). spFRET is also used to examine structural dynamics in large complexes, such as ribosomes. Several labeling strategies have been proposed to capture the dynamics of ribosomal conformation and the movements of bound tRNAs in the elongation phase of protein synthesis (Kapp and Lorsch 2004). Ferguson et al. (2015) measured changes in *E*_FRET_ over time between fluorescently labeled tRNA substrates that were bound in a human ribosome and found similalities and differences between bacterial and human ribosomes. For example, when a tRNA was bound within the peptidyl (P) site and a second tRNA was introduced to the aminoacyl (A) site, as in bacterial ribosomes, an intermediate-FRET state, in which GTP hydrolysis may take place, appeared after a transient low-FRET codon recognition state before the stable high-FRET bound state. However, the duration of the intermediate-FRET state was longer in human ribosomes, suggesting a distinct mechanism of tRNA selection. Another example is the thermodynamic stability that is observed in the POST complex, in which tRNAs are bound in the P and exit (E) sites, suggesting the existence of factors that are not required for bacterial ribosomes. Such distinct features of human ribosomes compared with bacterial ribosomes indicate a more complex regulation of protein synthesis.

## SMI of protein movements and interactions in cells

SMI allows one to visualize individual and collective molecular behaviors with spatiotemporal resolution in in vitro systems and living cells. Single proteins on the basal plasma membrane of cells that are attached to a glass substrate are observed with a good signal-to-noise ratio using total internal reflection illumination. SMI can even be performed with oblique illumination on the membrane of apical cell surface and cellular organelles, such as the nucleus (Tokunaga et al. 2008). The position and fluorescence intensity of each molecule can be determined in every frame of SMI. The frame-to-frame displacement of molecules is converted into the lateral diffusion coefficient or drift velocity, the values of which are often location-specific on the cell surface, reflecting a heterogeneous environment (Marguet et al. 2006). The fluorescence intensity that reflects the number of fluorescent molecules in each spot is used to quantify molecular clustering, which is often important for understanding molecular function (Iino et al. 2001; Ichinose et al. 2004; Ulbrich and Isacoff 2007). When state transitions in the movements or number of molecules are observed, the lifetime distribution of each state involves kinetic information such as the association and dissociation rate constants of molecular interaction (Hiroshima et al. 2012).

## Hidden Markov model analysis of protein movements and clustering

In most cases of SMI, the dynamics of molecular behaviors are analyzed in ensembles of single molecules (Lommerse et al. 2005; Hern et al. 2010). However, HMM, as used in spFRET analysis, is being used increasingly to analyze molecular movements (Chung et al. 2010; Low-Nam et al. 2011; Persson et al. 2013). HMM infers a discrete molecular state for every step in single-molecule trajectories; thus, it determines the real-time transition and real-space distribution of molecular states. HMM assumes that state transition occurs according to the probability matrix, *A*_*ij*_, where *i* and *j* are the states in the current and next frame, respectively. When local movements of the molecules are considered to be simple lateral diffusion, the distribution of the step displacement for the time interval, *δt*, obeys,

$$ P\left(r,t\right)=\sum \limits_{n=1}^N\frac{C_nr}{2{D}_n\delta t}\exp \left[\frac{-{r}^2}{4{D}_n\delta t}\right], $$

where *N* and *C*_*n*_ are the number of states and the fraction of state *n*, respectively. *D*_*n*_ is the diffusion coefficient of state *n*, and *r* is the displacement. The best fit values of these parameters can be estimated, for example, using the expectation–maximization algorithm (Dempster et al. 1977) for each model with a different number of states, and the most suitable number of states is determined by comparing the likelihoods for various state numbers, wherein a penalty for overfitting due to an increase in parameter species (state numbers) is considered. Trajectories of state transitions can be reconstructed, for example, using the Viterbi algorithm (Viterbi 1967).

Early applications of HMM analysis were on the examination of the movement of Q dot-labeled EGF receptor (EGFR), suggesting the existence of three states with different diffusion coefficients and state-specific mobility (Chung et al. 2010; Low-Nam et al. 2011). Each receptor state was believed to reflect movements in specific membrane structure, such as small membrane subdomains. On association of the ligand with EGFR, the diffusion became slower and more confined, possibly due to structural changes in EGFR that were induced by the ligand binding and/or phosphorylation on the cytoplasmic domain. A recent molecular dynamics (MD) simulation suggested a conformational change that could affect the interaction between an EGFR molecule and the membrane surface (Arkhipov et al. 2013).

HMM analysis is also applicable to the transitions in the number of molecules in a protein cluster. Dimerization of EGFR is needed for its phosphorylation and subsequent recognition by downstream signaling proteins, and further large oligomerization has also been reported (Clayton et al. 2005; Webb et al. 2008; Huang et al. 2016). Receptor clustering is reflected by an increase in the fluorescence intensity of single spots. To quantify the cluster size, stoichiometric labeling of the target protein is required. To this end, one such method is genetic fusion with a fluorescence tag, such as GFP, and its expression in cells that lack the endogenous protein. The sum of Gaussian distribution can be used as the observation probability in HMM molecular clustering analysis:

$$ \mathrm{P}\left(\mathrm{x}\right)=\sum \limits_n{C}_n\exp \left[-\frac{{\left(x- n\mu \right)}^2}{2n{\sigma}^2}\right]. $$

**Fig. 3:**
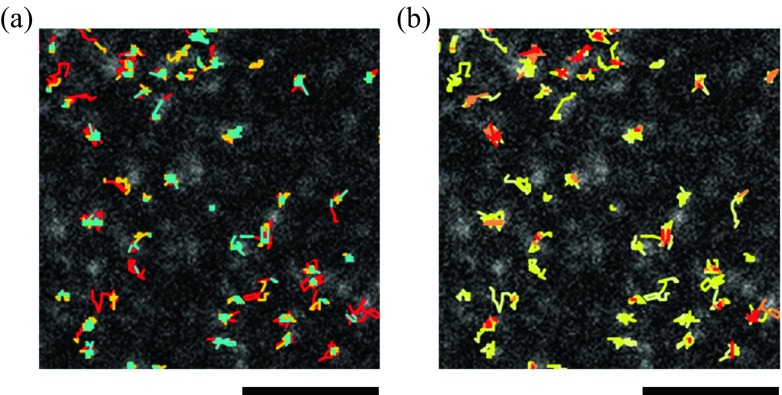
Spatial distribution of EGFR states along single-molecule trajectories for **a** diffusion and **b** clustering. Colors in (**a**) indicate three diffusion states: immobile (*blue*), slow diffusion (*orange*), and fast diffusion (*red*). In (**b**), cluster sizes from monomer to tetramer are colored from *yellow* to *red*. *Scale bar* 3 μm

Here, *x*, *μ*, *σ*, and *n* are the brightness of a spot, brightness of a single molecule, standard deviation for the brightness of single molecules, and cluster size, respectively, and *C*_*n*_ is the peak value of the cluster with size *n*. HMM analyses were performed simultaneously on the movements and clustering of EGFR-GFP that was expressed in EGFR-null CHO-K1 cells to identify a moderate correlation that the slowest state tended to be associated with a large cluster (Fig. 3). It is likely that cell signaling by EGFR is regulated by oligomerization beyond dimerization.

## SMI of protein interactions

Single-molecule analysis examines the structural mechanisms that underlie the kinetic properties of protein reactions. In contrast to the conventional kinetic measurements in ensemble molecules, SMI detects the start and end points of individual reaction events directly, and, thus, obtains reaction periods that are not obscured by, for example, the stochasticity that is caused by uncertain wait times for the collision of reactants, rendering SIM measurements suitable for detailed reaction analysis. In addition, because it is based on fluorescence detection, SMI measurement can be made *in cellula* (Nakamura et al. 2016; Yoshizawa et al. 2017).

**Fig. 4:**
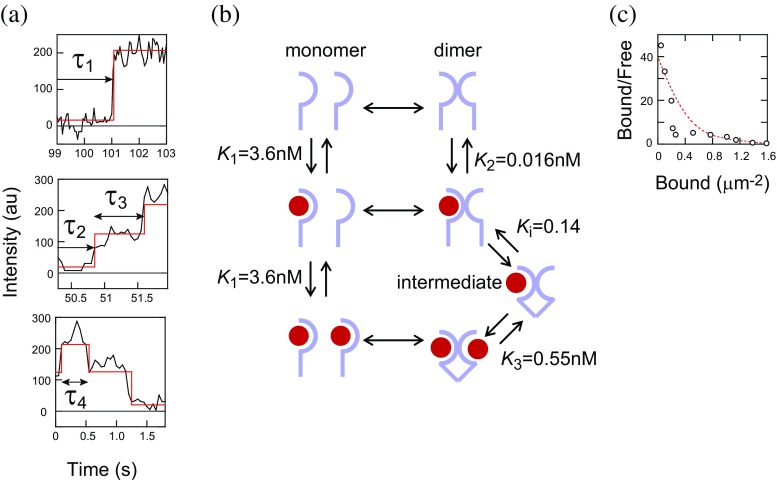
**a** Stepwise changes in the brightness of a single fluorescence spot. *Thick arrows* indicate associations or dissociation of fluorescent HRG. *τ*_1_–*τ*_3_ indicate wait times for association of HRG with receptor monomer, vacant receptor dimer, and receptor dimer with one ligand, respectively. *τ*_4_ indicates wait time for dissociation from receptor dimer with two ligands. **b** Reaction scheme of HRG association and receptor dimerization. An intermediate state exists between dimers with one and two ligands. Three dissociation equilibrium constants *K*_*1*_, *K*_*2*_, and *K*_*3*_, which depend on the receptor states, are indicated. **c** Scatchard plot for the association of HRG as an ensemble of molecules shows negative cooperativity. *Dotted line* was calculated based on the reaction model and parameters in (**b**)

The association of a cell differentiation factor, heregulin (HRG), with its receptors (HRGRs) and receptor dimerization were analyzed using SMI in living cells (Hiroshima et al. 2012). HRGRs, which are families of EGFR, form monomers and dimers on the cell surface. The association of a HRG that is labeled with a single fluorophore molecule was observed as the appearance of a fluorescent spot (a stepwise increase in fluorescence intensity) on the cell surface. During sparse ligand binding, the association of two HRG molecules at the same position indicates associations to HRGR dimers. For the first ligand associations to HGFR dimers, the distribution of wait times after the ligand was mixed with the culture medium showed a simple exponential decay. In contrast, the times between the first and second associations showed a distribution with a peak, indicating that an additional rate-limiting step exists prior to the second binding. As with the association, the dissociation of HRG from its receptors was detected as stepwise decreases in fluorescence intensity. The association and dissociation rate constants were obtained by fitting the time distributions using the appropriate chemical reaction models. There were three affinities between HRG and HRGRs. The highest affinity was found with vacant HRGR dimers, but association of the first ligand with vacant dimers decreased the affinity with the second ligand. The affinity with HRGR monomers was the lowest. Depletion of the highest affinity binding sites as the increase of ligand concentration results in a negative cooperativity. A structural change in HRGR dimers during the first and second association with HRG, observed as the additional rate-limiting step, caused negative cooperativity by increasing the dissociation rate constant (Fig. 4).

Extracellular signals to the EGFR family are transmitted into the cytoplasm to induce cell responses. The cytoplasmic proteins that recognize the phosphorylated EGFR family contain Src homology 2 (SH2) domains. Associations between EGFR and SH2 proteins have been investigated by SMI in cells (Jadwin et al. 2016). The temporal dynamics in the recruitment from the cytoplasm to the plasma membrane differ between SH2 proteins in living cells. Among SH2 proteins, GRB2 has a slower dissociation rate constant from EGFR clusters versus monomers. Multiplicity in the reaction rate constants between EGFR and GRB2 has also been observed in in vitro SMI measurement, wherein the association rate constant correlates inversely with the GRB2 concentration (Morimatsu et al. 2007). These results suggest that the cytoplasmic domain of EGFR binds GRB2 in multiple conformations and that the conformation bias depends on the size of the EGFR cluster.

## SMI of protein colocalizations and interactions

In-cell dual-color SMI allows one to compare the dynamics and kinetics between two molecular species. Ichinose et al. (2004) detected single molecules of EGF that were labeled with tetrametylrhodamine (TMR) and an active form of EGFR with Alexa488-labeled antibody to examine the association of EGF and activation of EGFR. Time-lapse dual-color SMI in semi-intact cells revealed that the density and cluster size of active EGFR molecules increased with time, exceeding those of EGF which induced the EGFR activation, meaning that an amplification of the EGF signal occurred through the dynamic EGFR clustering that will cause secondary activation of vacant receptor molecules.

Dynamic colocalization analysis of two protein species detects the kinetics of protein interactions. We have observed the interaction between GFP-tagged EGFR and a cytoplasmic protein GRB2 that had been tagged with TMR-labeled Halo protein in living cells. HMM is used to determine the colocalization periods, during which two differently colored spots overlap. The apparent reaction rate constant, as determined from the SMI measurements, must be corrected by the concentrations of labeled and unlabeled GRB2 molecules in the cytoplasm to obtain the true value. These GRB2 concentrations can be determined using FCS and western blot. The affinities with GRB2 differed for monomeric and clustered EGFR molecules, suggesting that a conformational change occurred after molecular clustering.

Another SMI method detects the extent of oligomerization of membrane proteins by counting the number of photo-bleaching steps in single fluorescent spots. GFP molecules that were fused to one subunit, two identical subunits, and two different subunits in a Ca^2+^ channel, CNG channel, and NMDA receptor, respectively, were used to measure the clusters in living cells (Ulbrich and Isacoff 2007). The stoichiometry of subunits in the channels/receptor was reflected by the number of bleaching steps counted. The fraction of non-fluorescent GFP was estimated to be ~20%, assuming binominal distribution of the photobleaching steps. This method can be applied to the quantitative analysis of other proteins with a biologically relevant cluster size and distribution.

## Super-resolution microscopy

Super-resolution microscopy techniques, such as photo-activated localization microscopy and stochastic optical reconstruction microscopy, determine the molecular positions with an accuracy of ten to several tens of nanometers, even for condensed molecules, such as those in protein clusters (Betzig et al. 2006; Rust et al. 2006). These techniques use images of momentarily discretized fluorescence emission from single molecules that are labeled with photo-convertible or photo-activable fluorescent probes, including mEOS, mKikGR, PA-mCherry, and PA-GFP (Lukyanov et al. 2005). Using super-resolution microscopy, a ring-like structure that was composed of actin–adducin complexes was clearly observed to be winding around axons with a periodicity of 180–190 nm, which is comparable with the size of spectrin tetramers that bridge the rings (Xu et al. 2013). Also, a localized pattern of chemotaxis-related protein clusters was resolved in *E.coli* cells allowing a mathematical model for chemotactic sensing to be constructed (Greenfield et al. 2009). The arrangement of gp120 in the nuclear pore complex was imaged as an eightfold symmetric structure with a central channel (Löschberger et al. 2012). As shown in these examples, super-resolution microscopy has been used to resolve protein arrangements that were unable to be determined using conventional crystallographic methods.

## Conclusion

Single-molecule fluorescence detection is useful for obtaining structural information on proteins and other biological macromolecules *in cellula* and in vitro. The greatest advantage of single-molecule measurements is that they provide information on structural distribution and dynamics. Having multiple conformations is a salient property of multi-domain proteins. In addition, single-molecule measurements allow one to perform a structural analysis of proteins and protein complexes without rigid structures, such as IDPs and dynamic protein clusters in cells. Because of often working as important HUB molecules in intracellular reaction networks for signal transduction, gene regulation, and metabolism, the dynamic structure–function relationship of these proteins and protein complexes in living cells is a highly interesting field.

Unfortunately, the structural information that single-molecule measurement provides is limited. Even using spFRET, which has a spatial resolution of 1 nm or less, only the distance and/or orientation between two locations on a molecule can be known. No atomic-level three-dimensional structure can be determined directly from single-molecule measurements. Cooperation with other technologies in structural analysis is indispensable. The development of novel methods to maximize the information from single-molecule measurements is ongoing. Data assimilation combining spFRET measurement and MD simulation (Matsunaga et al. 2015) may compensate for the poor spatial resolution in SMI. Multimodal measurements to enrich information, such as the simultaneous detection of polarization and fluorescence lifetime in spFRET measurement (Chung et al. 2016), will improve single-molecule structural biology. In-cell time series measurement with high temporal resolution may be another desirable technology. With the improvements in technologies, the roles of single-molecule measurements in structural biology are really increasing.
